# Content Validity Assessment of the Revised Illness Perception Questionnaire in CKD Using Qualitative Methods

**DOI:** 10.3390/ijerph19148654

**Published:** 2022-07-16

**Authors:** Eleanor Rivera, Kristin Levoy, Maya N. Clark-Cutaia, Sarah Schrauben, Raymond R. Townsend, Mahboob Rahman, James Lash, Milda Saunders, Rebecca Frazier, Hernan Rincon-Choles, Karen B. Hirschman

**Affiliations:** 1 Department of Population Health Nursing Science, College of Nursing, University of Illinois Chicago, Chicago, IL 60612, USA; 2 Department of Community and Health Systems, Indiana University School of Nursing, Bloomington, IN 47408, USA; klevoy@iu.edu; 3 Indiana University Center for Aging Research, Regenstrief Institute, Indiana University Melvin and Bren Simon Comprehensive Cancer Center, Indianapolis, IN 46202, USA; 4 Rory Meyers College of Nursing, New York University, New York, NY 10010, USA; mc7009@nyu.edu; 5 Division of Renal, Electrolyte, and Hypertension, Department of Biostatistics, Epidemiology, and Informatics, Perelman School of Medicine, University of Pennsylvania, Philadelphia, PA 19104, USA; Sarah.Schrauben@pennmedicine.upenn.edu (S.S.); townsend@upenn.edu (R.R.T.); 6 Division of Nephrology and Hypertension, Department of Medicine, University Hospitals Cleveland Medical Center, Case Western Reserve University, Cleveland, OH 44195, USA; Mahboob.Rahman@UHhospitals.org; 7 Division of Nephrology, College of Medicine, University of Illinois Chicago, Chicago, IL 60612, USA; jplash@uic.edu; 8 Department of Medicine, University of Chicago, Chicago, IL 60637, USA; msaunder@bsd.uchicago.edu; 9 Division of Nephorology and Hypertension, Feinberg School of Medicine, Northwestern University, Chicago, IL 60611, USA; rebecca.frazier@northwestern.edu; 10 Cleveland Clinic, Cleveland, OH 44195, USA; RINCONH@ccf.org; 11 Department of Biobehavioral Health Sciences, School of Nursing, University of Pennsylvania, Philadelphia, PA 19104, USA; hirschk@nursing.upenn.edu

**Keywords:** illness perceptions, chronic kidney disease, content validity, psychometric testing, illness perception questionnaire, illness representation

## Abstract

Background: The Revised Illness Perception Questionnaire (IPQ-R) measures individuals’ unique perceptions of their illness. While psychometric properties of the IPQ-R have been demonstrated in many disease populations, its content validity has not been extensively studied in non-dialysis chronic kidney disease (CKD). Unique features of CKD (e.g., few symptoms in early stages) may impact the measurement of illness perceptions. The purpose of this study was to explore the IPQ-R content validity in a sample of CKD patients. Methods: Thirty-one participants completed the IPQ-R and were interviewed regarding their subscale scores (timeline, consequences, personal control, treatment control, coherence, cyclical, and emotions). Participants’ agreement with their scores was tallied and assessed qualitatively for themes related to the content validity of the measure. Results: Individual participant agreement with their subscale scores averaged 79% (range: 29–100%). Subscale agreement varied: timeline (100%), consequences, coherence, and emotion (83% each), cyclical (75%), personal control (65%), and treatment control (64%). A qualitative exploration of disagreement responses revealed concerns with the relevance and comprehensibility of personal control and treatment control. Conclusions: Some IPQ-R subscales may pose content validity concerns in the non-dialysis CKD population. Item modification for comprehensibility (personal control) and relevance (treatment control) should be considered. Future studies should explore the impact of a patient’s symptom experience on IPQ-R validity, especially in populations like CKD with a higher proportion of asymptomatic patients.

## 1. Introduction

Illness perceptions are key components of the Common Sense Model of Illness Representations (CSM) [1]. The CSM posits that patients’ experiences with illnesses formulate perceptions in response to illness stimuli. Illness perceptions serve as important targets of intervention research, necessitating their accurate measurement. The Illness Perception Questionnaire (IPQ) has been widely used to assess illness perceptions since its development [2] and revision (IPQ-R) [3]. The IPQ-R assesses nine components of illness perceptions and produces a subscale for each component (timeline, consequences, personal control, treatment control, coherence, cyclical, emotion, identity, cause). Psychometric properties of the IPQ-R were demonstrated in a variety of disease populations during instrument development, including diabetes, multiple sclerosis, and myocardial infarction [3].

While the IPQ-R was designed to be used in any sample population without major modification, unique characteristics of chronic kidney disease (CKD) require an evaluation of content validity in this population [4]. Patients with early-stage CKD frequently have few to no symptoms, and treatment is often focused on active surveillance and management of upstream conditions such as hypertension and diabetes [5,6,7]. Some established IPQ-R constructs may not be relevant to the lived experience of patients with CKD. An in-depth exploration of CKD patients’ understanding of the IPQ-R constructs and their measurement is needed to determine if the IPQ-R adequately captures each component. The purpose of this study was to explore the content validity of the IPQ-R in a sample of CKD patients by assessing participants’ agreement with their IPQ-R results.

## 2. Materials and Methods

The sample for this study is composed of participants in the Chronic Renal Insufficiency Cohort Study (CRIC). CRIC is a prospective longitudinal CKD cohort study with participant sites nationwide; the inclusion/exclusion criteria, design, and baseline characteristics of CRIC have been described previously [8]. All CRIC participants have CKD stages 1–4 (i.e., no end-stage renal disease or dialysis) at the time of recruitment to the CRIC cohort, as determined by standard laboratory testing for glomerular filtration rate (GFR). In addition to the standard CRIC study criteria for this study, we also excluded participants with cognitive impairment, as determined by a score of <80 on the Modified Mini-Mental State Examination (3MS) [9]. The CRIC participants for this sample were all recruited from the CRIC site at the University of Pennsylvania, which was also the site of Institutional Review Board approval for this study. CRIC participants were given the option of participating in this supplemental study when they came in for their annual study visit. The sample size was determined when theoretical saturation on the topic was achieved [10].

After completing written informed consent for this study, all participants completed the 38-item IPQ-R on 7 out of 9 scale domains [3]. This study did not examine the identity or causes subscales because they do not have a consistent number of items nor averaged scores. Interviews were conducted via telephone 1 week to 2 months after IPQ-R data were collected (September 2019 to September 2020, all conducted by ER). At the start of the interview, participants’ results were read to them, one subscale at a time, asking structured questions such as, *“Your results show that you believe your CKD will last for a long time. Does that sound right to you?”* (i.e., timeline subscale), or, *“You believe that your treatment has a lot of control over how things are going. Does that sound right?”* (i.e., treatment control subscale). These questions were designed to restate the construct of each subscale while reflecting the individual’s specific score. Participants were encouraged to describe the accuracy of each subscale score based on their personal perceptions.

All interviews were recorded and professionally transcribed for analysis. Transcripts were independently reviewed (ER, KL) using a directed content analysis approach [11], coding the responses to each subscale as “agreed” or “disagreed” with the result. Any differences in coder determinations were discussed. Once consensus between coders was achieved, participants’ IPQ-R agreement rates for each subscale were tabulated. To add further explanatory insights into content validity, for all “disagreed” determinations, the coders used conventional content analysis [11] to explore patterns and themes. Statistical analysis for sample description and Cronbach’s alpha calculation were performed in STATA 16.

## 3. Results

Theoretical saturation was reached with a sample of 31 participants (15 men, 16 women) who completed both the IPQ-R and the subsequent interview about their scores. Their mean age was 67 years (range: 49–85 years). The racial breakdown among the 31 participants was 17 Black, 13 White, and 1 Hispanic/Latinx. Most had some college (*n* = 11) or a college degree or higher (*n* = 14). The mean glomerular filtration rate (GFR) captured at the time of IPQ-R data collection was 56.6 mL/min/1.73 m^2^ (Stage 3a CKD) and ranged from 18.7–92.4 (Stage 1–4) with a standard deviation of 18.7. Beck’s Depression Inventory (BDI) scores averaged 7.8, with 23 participants scoring with no depressive symptoms, 4 patients with mild depressive symptoms, and 4 patients with moderate to severe symptoms. Cronbach’s alpha reliability was calculated for all subscales: timeline (0.90), consequences (0.77), personal control (0.73), treatment control (0.66), coherence (0.86), cyclical (0.84), emotion (0.86).

Out of the 31 participants, six were in full agreement with their IPQ-R results, and the remaining 25 disagreed with at least one of their subscale results. The average participant agreement rate across all subscales was 79% (median: 86%; range: 29–100%). Subscales differed in their average agreement rates, where timeline had 100% agreement; consequences, coherence, and emotion each at 83%; cyclical (75%); and personal control and treatment control with the lowest agreement (65% and 64%, respectively) (see Table 1).

Overall, 43 responses among 25 participants had a “disagreed” determination. A qualitative exploration of the disagreement responses revealed two main themes—*inaccuracy* and *uncertainty* (Table 2). Inaccuracy was the more common disagreement theme (31 of 43 disagreement responses), with the remaining disagreement responses falling under uncertainty (12 of 43 disagreement responses). The inaccuracy theme reflected participants’ sentiments about the subscale that was different from the given score, such as, “*That’s not true*.” The *uncertainty* theme reflected that respondents did not perceive the subscale concept as relevant to their CKD experiences, with statements such as, “*I’m not sure how to answer that*” or *“I’ve never really thought about it”*. In the inaccuracy theme, almost 1/3 were associated with the personal control subscale (10 of 31 inaccuracy disagreement responses), with all participants reporting they felt that their personal control of their CKD was more limited than the IPQ-R score indicated. In the uncertainty theme, almost half of those that fell into this group were associated with the treatment control subscale (5 of 12 uncertainty disagreement responses). Overall, nearly half of all disagreement responses fell in the personal control and treatment control subscales (*n* = 21 of 43 disagreement responses).

## 4. Discussion

This is the first study to examine the content validity of the IPQ-R in a non-dialysis CKD population. At the patient level, the instrument captured overall illness perceptions perfectly in some patients (6/31), but participant perception agreement varied widely. While high agreement rates were detected with the timeline, consequences, coherence, cyclical, and emotion subscales, the frequent disagreements with the personal control and treatment control subscales present some content validity concerns. A qualitative exploration of these disagreements revealed either perception of score inaccuracy or uncertainty about the subscale content, which reflects issues with the comprehensibility and relevance dimensions of content validity [4].

There were the most disagreements with the personal control subscale, wherein a third of the sample disagreed with their results and these disagreements were almost all categorized as inaccuracy. This points to issues with comprehensibility: the personal control items may not accurately reflect the concept of personal control for CKD patients. During instrument development and psychometric testing, concurrent validity of the personal control subscale was demonstrated through association with coping behaviors in disease populations, including CKD patients [12]. However, this conceptualization of personal control as the performance of coping behaviors may not be aligned with CKD patient perceptions based on the wording of items. For example, the item “My actions will have no effect on the outcome of my kidney disease” may be interpreted differently or more broadly than just the concept of coping behaviors, including other factors such as treatment adherence (e.g., diet) and performance/avoidance of risky behaviors (e.g., cigarette smoking) [13]. Since most CKD patients experience a gradual decline in kidney function and may have received information from their healthcare providers to expect that illness pattern, they may have a different interpretation of the meaning of “my actions” or “outcome” with respect to their CKD than the IPQ-R authors intended. Effective communication between patients with CKD and their healthcare providers is a known issue with many barriers to success [14,15]. This comprehensibility issue could also extend to other chronic conditions with a similar trajectory, such as heart failure and chronic obstructive pulmonary disease.

Treatment control subscale measurement was complicated by participants who did not perceive that the concept was relevant to their CKD experience. Participants’ disagreements reflected uncertainty with the substance of the subscale items, and thus they were unable to respond appropriately. This may be a CKD-specific issue for the IPQ-R, as providers may not frame active surveillance and management of upstream conditions as a “treatment”. This is consistent with previous studies that have identified low levels of awareness and understanding of chronic kidney disease not only in patients but also in health care providers [16]. Therefore, the IPQ-R may not be measuring the theoretical construct as intended for CKD patients with respect to content validity relevance. These results point to potential issues for other conditions beyond CKD, such as hypertension, hyperlipidemia, early-stage diabetes, and other chronic illnesses with a low overall symptom profile and disease burden. Treatment control subscale items may need to be reworded or include examples with disease-relevant terms. Furthermore, the internal consistency reliability of treatment control was markedly lower than the other subscales (0.66), indicating further potential issues with this subscale.

There are some limitations to this study. Our sample size was not powered to make group-level comparisons for factors such as gender or race. We did not measure or assess validity for the identity or causes subscales because of their substantially different method of measurement and analysis from the included subscales. Another potential limitation is the difficulty in conveying or interpreting participant scores using plain language. Similarly, while all participants could speak and understand English, we did not explicitly assess fluency or whether English is their first or preferred language. Finally, there is some missing data due to participants who did not answer questions in a manner conducive to designating agreement/disagreement.

## 5. Conclusions

In conclusion, this study provides evidence to improve the content validity for several IPQ-R subscales for use in the early-stage CKD population. While most subscales held content validity, the personal control and treatment control subscales demonstrated issues with comprehensibility and relevance dimensions of content validity. These issues may warrant item modification. Such modifications may lead to a more accurate measurement of illness perceptions in this population and perhaps other disease populations with experiential similarities. Future studies should explore to what extent subjective symptom experience (i.e., whether patients experience symptoms or are asymptomatic) impacts the validity of the IPQ-R [17].

## Figures and Tables

**Table 1:** Individual IPQ-R scores and level of agreement across subscales.

Subject	Timeline	Consequences	Personal Control	Treatment Control	Coherence	Cyclical	Emotions	Individual Agreement (n/N)	% Agreement
1	4.3	**2.7 ^a^**	**4.0 ^a^**	4.0	4.0	2.0	2.3	5/7	71%
2	3.8	3.7	4.0	3.6 ^b^	3.6	3.8	3.8	6/6	100%
3	4.7	3.5 ^b^	**3.8 ^a^**	**3.2 ^a^**	3.4	**2.3 ^a^**	3.2	3/6	50%
4	4.0	2.5	3.3	4.0	2.0	**2.8 ^a^**	2.0	6/7	86%
5	2.8	**4 ^a^**	**3.7 ^a^**	3.4	2.4	3.3	3.2	5/7	71%
6	2.2	3.2	5.0	4.6	3.8	**3.0 ^a^**	2.5	6/7	86%
7	4.7	3.0	**3.8 ^a^**	**3.8 ^a^**	**2.8 ^a^**	**2.5 ^a^**	3.7	3/7	43%
8	3.2 ^b^	3.7	2.7	3.6	2.4	4.0	**3.5 ^a^**	5/6	83%
9	4.7	4.5	3.2	3.0	2.0 ^b^	2.5	**4.0 ^a^**	5/6	83%
10	4.2	2.7	3.5	**2.8 ^a^**	3.4	2.3	3.2	6/7	86%
11	4.5	3.2	**3.7 ^a^**	**3.6 ^a^**	2.4	1.8 ^b^	2.3 ^b^	3/5	60%
12	4.3	1.5	3.5	**3.2 ^a^**	2.8	2.0	1.0	6/7	86%
13	4.5	1.5	**4.7 ^a^**	4 ^b^	4.6	1.0	1.3	5/6	83%
14	4.3	3.3	5.0	5.0	**4.4 ^a^**	1.5	3.2	6/7	86%
15	4.0	**3 ^a^**	**4 ^a^**	3.6 ^b^	2.2 ^b^	**3.8 ^a^**	2.3	2/5	40%
16	4.0	3.3	3.7	3.2	3.6	2.0	2.0	7/7	100%
17	3.3 ^b^	**3 ^b^**	3.7	3.6	**3.6 ^a^**	3.0	**4.0 ^a^**	3/5	60%
18	3.0	2.7	3.3	4.0	2.2	3.3	2.3	7/7	100%
19	3.8	2.3	3.7	**3.6 ^a^**	4.0	2.5	2.0	6/7	86%
20	3.8	2.7	4.0	3.2	3.2	3.3	2.7	7/7	100%
21	3.7	3.3	4.0	4.0	3.2	3.5 ^b^	3.0	6/6	100%
22	4.0	3.3	4.0	**3.4 ^a^**	3.6	3.8	**2.3 ^a^**	5/7	71%
23	3.7	4.1	4.7	4.4	2.8	**2.5 ^a^**	3.3	6/7	86%
24	1.0	2.5	4.8	**4 ^a^**	2.8	2.5	2.2	6/7	86%
25	3.8	3.7	4.0	4.0	4.0	2.0	2.7	7/7	100%
26	4.0	3.0	4.0	3.6	**2.0 ^a^**	2.0	**2.7 ^a^**	5/7	71%
27	4.0	2.3	**3.7 ^a^**	3.2	4.0	2.3	2.3	6/7	86%
28	3.0	**2.3 ^a^**	**4 ^a^**	**4.6 ^a^**	**3.8 ^a^**	**3.5 ^a^**	1.3	2/7	29%
29	4.5	3.8	**4 ^a^**	**3.8 ^a^**	3.4	3.0	3.5	5/7	71%
30	4.2	4.3	**3.3 ^a^**	2.6	1.4	4.0 ^b^	2.2	5/6	83%
31	3.8	3.0 ^a^	4.0	3.6	2.0	3.5	4.0	6/7	86%
Total Agreement counts	29/29	24/29	20/31	18/28	24/29	21/28	25/30	161/204	
% agreement	100%	83%	65%	64%	83%	75%	83%	79%	

Note. Numbers indicate individuals’ IPQ-R scores for each subscale. If numbers are not bolded and have no superscripts next to them, the participant agreed that their score matched their CKD illness perceptions accurately for that subscale. Scores marked with superscript a (also bold font) indicate that the participant disagreed with their score. See Table 2 for further details on disagreements. Scores marked with superscript b indicate that the participant did not substantially engage with the question such that a designation of agreement or disagreement could be determined. Individual agreement denominators vary based on the number of responses that allowed for a determination, i.e., scores marked with superscript b were not included in the tally. Similarly, total agreement counts exclude any scores marked with superscript b.

**Table 2:** Themes Associated with Disagreement Responses.

Theme	IPQ-R Subscale					
	*Consequences*	*Personal Control*	*Treatment Control*	*Coherence*	*Cyclical Timeline*	*Emotions*
*Inaccuracy* of the score compared to their perception	“There are lots of consequences” (2.7) “I’m limited in what I can do” (3.0) “That is not true” (3.0)	“I don’t have an awful lot of control over it” (4) “A fair amount of control” (3.8) “Some control” (3.7) “I don’t think I have any control over it at all” (3.8) “I don’t think I got a lot of control” (3.7) “I think I have some control… I can only do so much” (4.7) “All the things that I should be doing, I can’t do” (4) “I don’t think I have a lot of control” (3.7) “I don’t think I have as much as I would like to think I have” (4.0) “Very little” (3.3)	“No” (3.8) “No” (3.6) “No” (3.4) “No, not really” (4.6) “A fair degree” (3.8)	“No I don’t” (2.8) “Somewhat” (4.4) “No not really” (3.6) “I think I have a good perspective of it” (2.0) “No, I don’t” (3.8)	“It has gone quite a bit lower from time to time” (2.3) “I feel like it’s the same day to day” (3.8) “I think it changes” (2.5)	“No… you just have to find a way to deal with it ” (3.5) “No” (4.0) “No” (4.0) “Well, I have to be angry” (2.3) “No” (2.7)
*Uncertainty* about their answer or lack of insight into the question	“I never thought about it” (2.3) “I wouldn’t say it was the kidney disease, it’s the fact that I had a stroke” (4)	“I’ve never really thought about it” (4.0)	“I’m not really having any treatment” (3.2) “Well, my treatment has been really just avoiding certain things.” (2.8) “My treatment is basically blood pressure meds.” (3.2) “I’m not really in treatment” (4.0) “Nothing’s been prescribed” (3.6)		“I really don’t know” (2.8) “I’m not sure how to answer that, I don’t know” (2.5) “Never thought about it” (3.5) “At this point, I have no way to measure to indicate how my kidney function is working” (3.0)	

Note: quotations are followed by the individual’s score on that subscale in parenthesis. Chronicity was not included because there was no disagreement for that subscale.

## Data Availability

The data presented in this study are available on request from the corresponding author. The data are not publicly available due to potential privacy concerns for patients regarding this qualitative data.
